# Information–Theoretic Radar Waveform Design under the SINR Constraint

**DOI:** 10.3390/e22101182

**Published:** 2020-10-20

**Authors:** Yu Xiao, Zhenghong Deng, Tao Wu

**Affiliations:** 1 School of Automation, Northwestern Polytechnical University, Xi’an 710072, China; dthree@nwpu.edu.cn; 2 Air and Missile Defense College, Air Force Engineering University, Xi’an 710051, China; 3 Equipment Management and UAV College, Air Force Engineering University, Xi’an 710051, China; taowu_nwpu@126.com

**Keywords:** information–theoretic, SINR constraint, waveform optimization, energy allocation strategies, frequency domain

## Abstract

This study investigates the information–theoretic waveform design problem to improve radar performance in the presence of signal-dependent clutter environments. The goal was to study the waveform energy allocation strategies and provide guidance for radar waveform design through the trade-off relationship between the information theory criterion and the signal-to-interference-plus-noise ratio (SINR) criterion. To this end, a model of the constraint relationship among the mutual information (MI), the Kullback–Leibler divergence (KLD), and the SINR is established in the frequency domain. The effects of the SINR value range on maximizing the MI and KLD under the energy constraint are derived. Under the constraints of energy and the SINR, the optimal radar waveform method based on maximizing the MI is proposed for radar estimation, with another method based on maximizing the KLD proposed for radar detection. The maximum MI value range is bounded by SINR and the maximum KLD value range is between 0 and the Jenson–Shannon divergence (J-divergence) value. Simulation results show that under the SINR constraint, the MI-based optimal signal waveform can make full use of the transmitted energy to target information extraction and put the signal energy in the frequency bin where the target spectrum is larger than the clutter spectrum. The KLD-based optimal signal waveform can therefore make full use of the transmitted energy to detect the target and put the signal energy in the frequency bin with the maximum target spectrum.

## 1. Introduction

Cognitive radar can be used to adaptively investigate the radar scene and determine following actions based on previous measurements. Transmitted waveform design is an important factor affecting radar system performance, which is attracting increasing attention [1,2,3,4]. One of the most important aspects of signal waveform design is the choice of an optimization metric. Many metrics were proposed for radar waveform optimization, such as the mutual information (MI) [5], the minimum mean squared error (MMSE) [6,7], the Kullback–Leibler divergence (KLD) [8], and signal-to-noise ratio (SNR) [9,10]. Our focus in this work is on designing waveforms based on information–theoretic metrics to maximize target estimation and detection performance. Bell [11] first proposed that information–theoretic tools are important for radar waveform design and considered the problems of optimal target detection and optimal target information extraction.

In the frequency-domain waveform design approach, much of the existing waveform design literature addresses designs that consider the energy constraint. In [12], different cases of the Lagrangian multiplier search region in the water-filing waveform proposed by Bell [11] were discussed. The essence of this problem is the energy constraint. In [13], the MI-based waveform design in signal-dependent interference and the MI-SNR relationship were investigated, and a comprehensive theory of matched illumination waveforms for extended targets was presented. Considering the uncertainties of radar target prior information, signal-to-interference-plus-noise ratio (SINR)- and MI-based maximin robust waveforms were designed in [14]. By defining mutual information based on energy spectral variance, a classification waveform algorithm was derived in [15]. These waveform design methods all aim to distribute the limited energy over the same bands as the expected target. With the development of electronic countermeasure technology, waveform design presents a need to solve radar interference issues. In [16,17], the technique from [11] was extended to a target defense application, and task-dependent, power-constraint, optimal jamming techniques were investigated; the main goal was to minimize the MI between the radar echoes and the stochastic target impulse response. In addition, the waveform strategy design problem for the jammer based on MI was considered in [18]. Considering the benefits of MI analysis, we herein utilize the MI between the target response and the target echoes as the waveform design metric.

Another information–theoretic metric used to maximize target detection performance is the KLD, which is appropriate for radar waveform design when the signal is small [19,20,21,22]. From a detection point of view, the more appropriate and proportionate solution is the KLD between the probability distributions of the observations under the two hypotheses [23,24,25,26]. Moreover, in [23,24], it was shown that the larger the relative entropy was, the better the obtained detection performance. In [27], the signal waveform was optimized to maximize the detection performance of the locally most powerful (LMP) detector or, equivalently, the KLD. In [28], the authors considered several popular information–theoretic criteria with similar characteristics, and a unified framework using of the Bhattacharyya distance, the KLD, the Jenson–Shannon divergence (J-divergence), and MI was proposed to describe all of the arising waveform optimization strategies. As shown in [19], this explained the tradeoffs of the KLD, MI, and SNR metrics currently used for waveform design. Furthermore, the relationship KLD = SNR − MI connects the three metrics in extended target waveform design. However, it should be noted that the above studies focused on the waveform design under energy constraints.

From the above discussion, MI and KLD criteria were clearly the primary metrics used to design radar waveforms. The optimal radar waveform is highly mission-dependent and depends on the target and surrounding environments; hence, a change in the target and environment causes the signal-to-interference-plus-noise (SINR) to change. However, less attention was paid to waveform design methods in the case of existing SINR constraints [29,30,31]. To our knowledge, a novel optimal radar waveform design method that combines the SNR and MI criteria was studied in [32], providing a smooth trade-off between the SNR of a particular target and the MI between the target response and the target echoes. Nevertheless, the SNR value range should be considered in waveform design. In this paper, we hope to model and solve the trade-off among MI, the KLD, and the SINR and provide a waveform design method based on information theory. In [19], the direct relationship between three measurement metrics, MI, the KLD, and SINR, was proposed, providing us with a way to verify the theoretic radar waveform design under the SINR constraint.

The primary innovations of our work are summarized as follows:

(1) Based on the nonnegativity of relative entropy, a model constraint relationship between the MI, the KLD, and the SINR is established in the frequency domain. It may be inferred that the maximum MI value range is bounded by SINR, and the maximum KLD value range is between 0 and the J-divergence value.
(2) The effect of the SINR value range on maximizing MI and KLD under the energy constraint is derived, which is considered in the presence or absence of clutter, respectively.
(3) Under the constraints of energy and SINR, a radar waveform optimal method based on maximizing MI and maximizing KLD is proposed.

The organization of this paper is as follows. In Section 2, we describe the target echo signal model and the binary hypothesis problem. In Section 3, we derive the value range of the MI and the KLD metric and present the constraint relationship between the MI and the KLD with the SINR. In Section 4, we present MI- and KLD-based optimal waveform designs under the SINR constraint as well as the SINR value range. The simulation results demonstrating the proposed scheme are presented in Section 5. Finally, our conclusions and directions for possible future work are drawn in Section 6.

## 2. Signal Model

For this work, we considered the waveform design of an active radar system in the presence of signal-dependent clutter. Specifically, we revisited the problem and found a frequency domain expression for the transmitted waveform. We used the system model detailed in Romero et al. [13] as our basic starting point. Let h(t) be a known complex-valued baseband target impulse response of finite duration $T_{h}$ and Fourier transform H(f). Let c(t) be a complex-valued, zero-mean, Gaussian random process representing a clutter component and characterized by the power spectral density (PSD) $P_{c}\left(f\right)$. Let n(t) be an independent, zero-mean, Gaussian random variable with variance $\sigma_{n}^{2}$. In a general transmitted waveform scenario, a radar transmitter transmits a waveform and the receiver receives the echo through the reflection of the environment and the target. Consider x(t) to denote the transmitted waveform. The expression of the target echo signal y(t) can be denoted as [13]

(1) y(t)=h(t)∗x(t)+c(t)∗x(t)+n(t)

where * denotes the convolution operator, and x(t) is a finite-energy waveform with bandwidth $W=\left[f_{0}\;,\;f_{0}+w\right]$ and spectrum response X(f). Then, the transmitted waveform’s energy is

(2) $$E_{x}=\int_{-\infty }^{\infty }{\left|X(f)\right|}^{2}df$$

The basic problem of target detection is choosing one of the two possible hypotheses using as few observations as possible while maintaining the error rates of both hypotheses below a predefined level. According to the signal model mentioned above, the target detection problem can be cast as the following binary hypothesis problem:

(3) $$\begin{array}{l} H_{0}\;:\;y(t)=c(t)*x(t)+n(t)\\ H_{1}\;:\;y(t)=h(t)*x(t)+c(t)*x(t)+n(t) \end{array}$$

where hypothesis $H_{0}$ indicates that there is only clutter and noise in the echoes, and hypothesis $H_{1}$ denotes that the radar echoes contain the target, clutter, and noise.

The energy spectral variance (ESV) describes the average energy of a finite-duration, zero-mean process; it is the same as power spectral density (PSD) and describes the average power of an infinite-duration, wide-sense, stationary process [11]. Since h(t) is a finite-energy process, the target ESV can be formulated as $\sigma_{h}^{2}(f)=E\left[{\left|H(f)-\mu_{h}(f)\right|}^{2}\right]$, where $\mu_{h}(f)=E[H(f)]$ is the mean of the random transfer function. In this paper, assuming that the target model is Gaussian, we obtain $\mu_{h}(f)=0$, in which case the target energy spectral density and ESV functions are equal.

Assume that the transmitted signal energy is constrained to be $\int_{W}{\left|X(f)\right|}^{2}df\leq E_{x}$ and that ${\left|X(f)\right|}^{2}$ is the energy spectral density (ESD). Then, the detection problem of (3) is equivalent to

(4) $$\begin{array}{l} H_{0}\;:\;Y(f)=P_{c}(f){\left|X(f)\right|}^{2}+\sigma_{n}^{2}\\ H_{1}\;:\;Y(f)=\sigma_{h}^{2}(f){\left|X(f)\right|}^{2}+P_{c}(f){\left|X(f)\right|}^{2}+\sigma_{n}^{2} \end{array}$$

The Neyman Pearson (NP) approach is the classical way of solving detection problems in an optimal manner. However, the exact closed-form probability density function (PDF) of the NP detector is hard to derive, even with the large sample, frequency-domain approach we took. This may have motivated the information–theoretic approach to signal design. Consequently, we assumed a priori knowledge about the target ESV, clutter PSD, and noise variance, which was justified using the information of the previous scans [33,34]. Based on a certain number of received data, one can decide whether a target is present or not. Thus, the function in (4) shows that waveform ESD ${\left|X(f)\right|}^{2}$ represents a degree of freedom in the design of the detection architecture. However, under the conditions of a given transmitted power constraint and SINR constraint, the resulting optimum waveform is highly dependent on all three spectra and constraints. In the next section, we resort to an information–theoretic approach to address the constraints and waveform design problem.

## 3. A Relationship among the MI, KLD, and SINR

We can use MI, the KLD, and the SINR as metrics of radar waveform design, but there is a relationship among all three terms. From previous work [13,19], we observed that MI-, KLD-, and SINR-based waveforms form the transmission spectra differently. Evaluating the relationship among the MI, KLD, and SINR for waveform design allowed us to derive the mathematical relationship on how these waveforms are formed.

### 3.1. MI and KLD Value Range

The relative entropy or the KLD is a measure of the distance between two distributions [35]. There are many PDF distance measures used to quantify the disparity between PDFs, such as the KLD, the Bhattacharyya distance, and the J-divergence. Among these distance measures, the KLD was confirmed to be a powerful and accurate tool to measure the information of multivariate data with lower complexity and superior performance [20]. Let $D(p_{0}(Y)∥p_{1}(Y))$ denote the relative entropy between hypotheses $H_{0}$ and $H_{1}$, where $p_{0}(Y)$ and $p_{1}(Y)$ are the PDFs of Y(f) under hypotheses $H_{0}$ and $H_{1}$, respectively. For brevity, we denote $D(p_{0}(Y)∥p_{1}(Y))$ by $D(p_{0}∥p_{1})$. Like the relative entropy between hypotheses $H_{0}$ and $H_{1}$, let $D(p_{1}∥p_{0})$ be the relative entropy between hypotheses $H_{1}$ and $H_{0}$. With a large data record assumption (i.e., as N→∞), the asymptotic KLD between two multivariate, Gaussian PDFs can be shown as [19]

(5) $$D(p_{0}∥p_{1})=\frac{N}{2}\int_{W}\left[\frac{P_{0}(f)}{P_{1}(f)}-\ln\frac{P_{0}(f)}{P_{1}(f)}-1\right]\;df$$

(6) $$D(p_{1}∥p_{0})=\frac{N}{2}\int_{W}\left[\frac{P_{1}(f)}{P_{0}(f)}-\ln\frac{P_{1}(f)}{P_{0}(f)}-1\right]\;df$$

According to the PDFs of Y(f) under hypotheses $H_{0}$ and $H_{1}$ given by (4), the relative entropy can be straightforwardly obtained as follows:

(7) $$D(p_{0}∥p_{1})=\frac{N}{2}\int_{W}\{\ln\left[1+\frac{\sigma_{h}^{2}(f){\left|X(f)\right|}^{2}}{P_{c}(f){\left|X(f)\right|}^{2}+\sigma_{n}^{2}}\right]-\;\frac{\sigma_{h}^{2}(f){\left|X(f)\right|}^{2}}{\sigma_{h}^{2}(f){\left|X(f)\right|}^{2}+P_{c}(f){\left|X(f)\right|}^{2}+\sigma_{n}^{2}}\}df$$

(8) $$D(p_{1}∥p_{0})=\frac{N}{2}\int_{W}\{\frac{\sigma_{h}^{2}(f){\left|X(f)\right|}^{2}}{P_{c}(f){\left|X(f)\right|}^{2}+\sigma_{n}^{2}}-\ln\left[1+\frac{\sigma_{h}^{2}(f){\left|X(f)\right|}^{2}}{P_{c}(f){\left|X(f)\right|}^{2}+\sigma_{n}^{2}}\right]\}df$$

Hence, by the nonnegativity of relative entropy [35], i.e., $D(p_{0}∥p_{1})\geq 0$ and $D(p_{1}∥p_{0})\geq 0$, we obtain

(9) $$\int_{W}\frac{\sigma_{h}^{2}(f){\left|X(f)\right|}^{2}}{\sigma_{h}^{2}(f){\left|X(f)\right|}^{2}+P_{c}(f){\left|X(f)\right|}^{2}+\sigma_{n}^{2}}\;df\leq \;\int_{W}\ln\left[1+\frac{\sigma_{h}^{2}(f){\left|X(f)\right|}^{2}}{P_{c}(f){\left|X(f)\right|}^{2}+\sigma_{n}^{2}}\right]\;df\leq \;\int_{W}\frac{\sigma_{h}^{2}(f){\left|X(f)\right|}^{2}}{P_{c}(f){\left|X(f)\right|}^{2}+\sigma_{n}^{2}}\;df$$

For conciseness of notation, the following is defined as:

(10) $$\mathrm{XINR}=\int_{W}\frac{\sigma_{h}^{2}(f){\left|X(f)\right|}^{2}}{\sigma_{h}^{2}(f){\left|X(f)\right|}^{2}+P_{c}(f){\left|X(f)\right|}^{2}+\sigma_{n}^{2}}\;df$$

(11) $$\mathrm{SIN}R(f)=\frac{\sigma_{h}^{2}(f){\left|X(f)\right|}^{2}}{P_{c}(f){\left|X(f)\right|}^{2}+\sigma_{n}^{2}}$$

For the extended random target case, a related result is used to describe the MI and the SINR relationship [13], i.e., the MI is related to the SINR via

(12) $$\mathrm{MI}=\int_{W}\ln\left[1+\mathrm{SIN}R(f)\right]df$$

Then, we can rewrite the XINR expression as

(13) $$\mathrm{XINR}=\int_{W}\left[\frac{\mathrm{SIN}R(f)}{1+\mathrm{SIN}R(f)}\right]\;df$$

The MI in (9) may therefore be rewritten in a more simplified form:

(14) XINR≤MI≤SINR

The interesting relationship (14) between the MI, the XINR, and the SINR provides useful insight into the waveform design problem. As shown, the MI and the XINR are functions of the SINR spectral density. Clearly, for the known SINR spectral density case, the transmitted waveform design may be derived via inspection of $\int_{W}\mathrm{SIN}R(f)\;df$, which gives the minimum and the maximum of the MI.

Furthermore, as in [19,28], the KLD associated with (4) can be obtained as $D(p_{0}∥p_{1})=\mathrm{MI}-\mathrm{XINR}$, which connects the three waveform design metrics in the extended target waveform design. We already know from (14) that MI is related to SINR and XINR, which leads to the KLD described by

(15) 0≤KLD≤SINR-XINR

In fact, the J-divergence [21] can be expressed as $\mathrm{Div}=D(p_{0}∥p_{1})+D(p_{1}∥p_{0})$. By using (7) and (8), the J-divergence can be equivalently rewritten as

(16) $$D(p_{0}∥p_{1})+D(p_{1}∥p_{0})=\mathrm{SIN}R-\mathrm{XINR}$$

Therefore, the KLD can be recast as the following:

(17) 0≤KLD≤J-divergence

Indeed, the KLD and the J-divergence are also functions of the SINR spectral density. In the following, we provide a useful remark discussing the constraint relationships between the MI and the KLD with the SINR.

### 3.2. Constraints between the MI and KLD with SINR

It was shown in [32] that the designed waveform approach satisfies both the SNR constraint and the maximum MI objective. A large SNR margin beyond the SNR value range does not contribute to target detection; therefore, a waveform that can carry more information about the target based on the satisfaction of its SNR detection threshold can be simplified. However, less attention is paid to discussing the constraints between MI and KLD with SINR and the SINR detection threshold value ${\mathrm{SIN}R}_{0}$ for the receiver in the presence of clutter. To gain insight into how SINR affects MI and KLD, we used the following expressions.

From the MI expression (12), let MI(f) be the MI spectral density, given by

(18) $$\mathrm{MI}(f)=\int_{0}^{f}\ln\left[1+\mathrm{SIN}R(f)\right]\;df$$

Similarly, the expression of KLD spectral density KLD(f) can be denoted by

(19) $$\mathrm{KLD}(f)=\int_{0}^{f}\ln\left[1+\mathrm{SIN}R(f)\right]-\frac{\mathrm{SIN}R(f)}{1+\mathrm{SIN}R(f)}\;df$$

According to (18) and (19), the MI and the KLD spectral density are functions of the SINR spectral density. Note that in (11), the SINR spectral density SINR(f) is nonnegative, therefore the MI and the KLD spectral density are always nonnegative (i.e., MI(f)≥0 and KLD(f)≥0). Therefore, the MI and the KLD spectral density change with the SINR spectral density. The SINR(f), however, is not a monotonic function of frequency f and can adapt effectively to the changing target and environments. Hence, the MI- and KLD-based waveform designs should consider the SINR constraints. In particular, the achievable SINR is smaller than the detecting threshold when the target is lost in a cluttered environment, which may have a large effect on the formation of these waveforms. On the other hand, the achievable SINR region under the transmitted energy constraint is bounded, and the achievable energy may have an effect on the formation of these waveforms when the SINR reaches a certain level.

## 4. Information–Theoretic Optimal Radar Waveform Design

To achieve greater detection performance, the radar should transmit the waveform matched with the environment to illuminate the target. We considered the information-based, waveform solution approach in the presence of signal-dependent clutter, as discussed herein. The problem of optimal waveform spectrum design was formulated based on the maximum MI criterion, maximum KLD criterion, and a trade-off between the MI, KLD, and SINR. Then, the SINR value range was investigated.

### 4.1. SINR Constraint Formulation

According to (1), the received echo signal is the sum of the desired signal component h(t)∗x(t), the unwanted clutter component c(t)∗x(t), and the noise component n(t), whose transmitted energies are $E_{signal}$, $E_{clutter}$, and $E_{noise}$, respectively. Therefore, the SINR can be expressed as

(20) $$\mathrm{SIN}R=\frac{E_{signal}}{E_{clutter}+E_{noise}}$$

where the energy of the target signature signal is

(21) $$E_{signal}=\int_{0}^{T_{y}}{\left|h(t)*x(t)\right|}^{2}\;dt=\int_{0}^{T_{h}}h(\tau_{1})\int_{0}^{T_{h}}h(\tau_{2})R_{xx}(\tau_{2}-\tau_{1})\;d\tau_{2}d\tau_{1}$$

The autocorrelation function of the transmitted signal can be defined as $R_{xx}(t)=\int x(t)x(t+\tau )\;dt$.

We can also obtain the energy of the clutter signal as

(22) $$E_{clutter}=\int_{0}^{T_{h}}c(\tau_{1})\int_{0}^{T_{h}}c(\tau_{2})R_{xx}(\tau_{2}-\tau_{1})\;d\tau_{2}d\tau_{1}$$

Now, by taking into account (21) and (22), using a sampling interval $T_{s}$ to discretize $E_{signal}$ and $E_{clutter}$ [32], we can rewrite SINR as

(23) $$\mathrm{SIN}R=\frac{T_{s}^{2}\left[H^{T},0_{1\times (L_{x}-L_{h})}\right]R_{xx}}{T_{s}^{2}\left[C^{T},0_{1\times (L_{x}-L_{h})}\right]R_{xx}+\sigma_{n}^{2}}$$

where

(24) $$H^{T}=h^{T}\left[\begin{array}{ll} h[1]\;2h[2]\;\cdots \;2h[L_{h}-1] & 2h[L_{h}]\\ h[2]\;2h[3]\;\cdots \;2h[L_{h}] & \;\;0\\ \;\;\;\vdots \;\;\;\;\;\;\vdots \;\;\;\;\;\;\;\;\;\;\;\;\;\vdots & \;\;\vdots \\ h[L_{h}]\;\;0\;\;\;\;\;\;\;\;\;\;\;\;0 & \;\;0 \end{array}\right]$$

(25) $$C^{T}=c^{T}\left[\begin{array}{ll} c[1]\;2c[2]\;\cdots \;2c[L_{h}-1]\; & 2c[L_{h}]\\ c[2]\;2c[3]\;\cdots \;2c[L_{h}] & \;\;0\\ \;\;\;\vdots \;\;\;\;\;\;\vdots \;\;\;\;\;\;\;\;\;\;\;\;\;\vdots & \;\;\vdots \\ c[L_{h}]\;\;0\;\;\;\;\;\;\;\;\;\;\;\;0 & \;\;0 \end{array}\right]$$

with $h={\left[h[1]\;h[2]\;\cdots \;h[L_{h}-1]\;\;h[L_{h}]\;\right]}^{T}$, $c={\left[c[1]\;c[2]\;\cdots \;c[L_{h}-1]\;\;c[L_{h}]\;\right]}^{T}$, and $R_{xx}$=${\left[R_{xx}[0]\;R_{xx}[1]\;\cdots \;R_{xx}[n]\;\right]}^{T}$.

Therefore, if we set up an SINR detection threshold ${\mathrm{SIN}R}_{0}$ for the receiver, the SINR constraint can be expressed by

(26) $$\frac{T_{s}^{2}}{\sigma_{n}^{2}}\left[\left[H^{T},0_{1\times (L_{x}-L_{h})}\right]-\left[C^{T},0_{1\times (L_{x}-L_{h})}\right]{\mathrm{SIN}R}_{0}\right]R_{xx}\leq {\mathrm{SIN}R}_{0}$$

Note that (26) is an inequality regarding the transmitted signal autocorrelation function $R_{xx}$, not ESD ${\left|X(f)\right|}^{2}$. In this case, using the Wiener Khinkin theorem, the ESD of x(t) can be calculated using $R_{xx}$, i.e.,

(27) $${\left|X(f)\right|}^{2}=R_{xx}[0]+2\overset{L_{x}-1}{\underset{n=1}{\Sigma }}R_{xx}[n]\cos(2\pi nf)\;=\psi_{f}^{T}R_{xx}$$

where

(28) $$\psi_{f}={\left[1,2\cos(2\pi f),2\cos(4\pi f),\ldots ,\cos(2\pi (L_{x}-1)f)\right]}^{T}$$

By setting $S_{xx}={\left[{\left|X(f_{1})\right|}^{2}\;{\left|X(f_{2})\right|}^{2}\;\cdots \;{\left|X(f_{N})\right|}^{2}\right]}^{T}$ and $\psi ={\left[\psi_{f_{1}}\;\psi_{f_{2}}\;\cdots \;\psi_{f_{N}}\right]}^{T}$, the transmitted signal ESD vector can be described using the following model:

(29) $$S_{xx}=\psi R_{xx}$$

where $R_{xx}$ can be solved using the Moore Penrose generalized matrix inverse, i.e., $R_{xx}=\psi^{†}S_{xx}$.

Therefore, the SINR constraint (26) can be expressed by

(30) $$\frac{T_{s}^{2}}{\sigma_{n}^{2}}\left[\left[H^{T},0_{1\times (L_{x}-L_{h})}\right]-\left[C^{T},0_{1\times (L_{x}-L_{h})}\right]{\mathrm{SIN}R}_{0}\right]\psi^{†}S_{xx}\leq {\mathrm{SIN}R}_{0}$$

### 4.2. Waveform Design Using Mutual Information

To enhance the detection performance of extended targets, we considered waveform optimization to maximize the MI, a problem originally posed by Bell [11] that led to the use of MI as a waveform design criterion. In the presence of clutter, the expression of the approximate MI based on the signal model in (4) is shown as

(31) $$\mathrm{MI}\left({\left|X(f)\right|}^{2}\right)=\int_{W}\ln\left[1+\frac{\sigma_{h}^{2}(f){\left|X(f)\right|}^{2}}{P_{c}(f){\left|X(f)\right|}^{2}+\sigma_{n}^{2}}\right]\;df$$

#### 4.2.1. Without SINR Constraints

To improve the performance of target detection, the designed optimal transmitted waveform under the energy constraint should satisfy [13]

(32) $$\begin{array}{l} \underset{{\left|X(f)\right|}^{2}}{\max}\;\;\int_{W}\ln\left[1+\frac{\sigma_{h}^{2}(f){\left|X(f)\right|}^{2}}{P_{c}(f){\left|X(f)\right|}^{2}+\sigma_{n}^{2}}\right]\;df\\ s.t.\;\int_{W}{\left|X(f)\right|}^{2}\;df\leq E_{x} \end{array}$$

The above constraint optimization problem can be solved by the method of Lagrangian multipliers as follows [13]:

(33) $${\left|X_{\mathrm{MI}-\mathrm{NoSINR}}\left(f\right)\right|}^{2}=\max\left\{0,B(f)\left[A-D(f)\right]\right\}$$

where

(34) $$B(f)=\frac{\sigma_{h}^{2}\left(f\right)}{2P_{c}\left(f\right)+\sigma_{h}^{2}\left(f\right)}$$

(35) $$D(f)=\frac{P_{n}\left(f\right)}{\sigma_{h}^{2}\left(f\right)}$$

and A denotes a constant that can be derived by the constraint of energy

(36) $$\int_{W}\max\left\{0,B(f)\left[A-D(f)\right]\right\}\;df\leq E_{x}$$

The results show that the spectrum response of the optimal waveform solution based on MI can be obtained using the water-filling algorithm.

#### 4.2.2. With SINR Constraints

Under the SINR and energy constraints, the MI-based radar waveform design problem can be formulated as the following optimization problem:

(37) $$\begin{array}{l} \underset{{\left|X(f)\right|}^{2}}{\max}\;\;\int_{W}\ln\left[1+\frac{\sigma_{h}^{2}(f){\left|X(f)\right|}^{2}}{P_{c}(f){\left|X(f)\right|}^{2}+\sigma_{n}^{2}}\right]\;df\\ s.t.\;\int_{W}{\left|X(f)\right|}^{2}\;df\leq E_{x}\\ \;\;\;\;\frac{T_{s}^{2}}{\sigma_{n}^{2}}\left[\left[H^{T},0_{1\times (L_{x}-L_{h})}\right]-\left[C^{T},0_{1\times (L_{x}-L_{h})}\right]{\mathrm{SIN}R}_{0}\right]\psi^{†}S_{xx}\leq {\mathrm{SIN}R}_{0}\\ \;\;\;\;S_{xx}={\left[{\left|X(f_{1})\right|}^{2}\;{\left|X(f_{2})\right|}^{2}\;\cdots \;{\left|X(f_{N})\right|}^{2}\right]}^{T} \end{array}$$

To solve this problem, we recognized that the transmitted waveform is constrained according to (37) and that the integration kernel of (31) is concave, leading to the use of the Lagrangian multiplier technique. To obtain a convenient expression for the objective of the design problem, we can define

(38) $$\beta =[\beta_{f_{1}}\;\beta_{f_{2}}\;\cdots \;\beta_{f_{N}}]=\frac{T_{s}^{2}}{\sigma_{n}^{2}}\left[\left[H^{T},0_{1\times (L_{x}-L_{h})}\right]-\left[C^{T},0_{1\times (L_{x}-L_{h})}\right]{\mathrm{SIN}R}_{0}\right]\psi^{†}$$

Using (38), the SINR constraint in (37) can be rewritten as

(39) $$\int_{W}\beta_{f}{\left|X(f)\right|}^{2}\;df\leq {\mathrm{SIN}R}_{0}$$

The optimization problem in (37) can be reformulated as

(40) $$\begin{array}{l} \underset{{\left|X(f)\right|}^{2}}{\max}\;\;\int_{W}\ln\left[1+\frac{\sigma_{h}^{2}(f){\left|X(f)\right|}^{2}}{P_{c}(f){\left|X(f)\right|}^{2}+\sigma_{n}^{2}}\right]\;df\\ s.t.\;\int_{W}{\left|X(f)\right|}^{2}\;df\leq E_{x}\\ \;\;\;\;\int_{W}\beta_{f}{\left|X(f)\right|}^{2}\;df\leq {\mathrm{SIN}R}_{0} \end{array}$$

The Lagrangian multiplier technique can therefore be utilized, and the objective function can be yielded as

(41) $$\mu ({\left|X(f)\right|}^{2},\lambda_{1},\lambda_{2})=\ln\left[1+\frac{\sigma_{h}^{2}(f){\left|X(f)\right|}^{2}}{P_{c}(f){\left|X(f)\right|}^{2}+\sigma_{n}^{2}}\right]-\lambda_{1}({\left|X(f)\right|}^{2}-E_{x})-\lambda_{2}(\beta_{f}{\left|X(f)\right|}^{2}-{\mathrm{SIN}R}_{0})$$

Taking the partial derivative of $\mu ({\left|X(f)\right|}^{2},\lambda_{1},\lambda_{2})$ with respect to ${\left|X(f)\right|}^{2}$ and setting it to zero, we obtain

(42) $$\lambda_{1}+\lambda_{2}\beta_{f}=\frac{\sigma_{h}^{2}(f)P_{n}(f)}{A(f){\left|X(f)\right|}^{4}+B(f){\left|X(f)\right|}^{2}{+P}_{n}^{2}(f)}$$

where

(43) $$A(f{)=P}_{c}(f)\left[P_{c}(f)+\sigma_{h}^{2}(f)\right]$$

(44) $$B(f{)=P}_{n}(f)\left[2P_{c}(f)+\sigma_{h}^{2}(f)\right]$$

By setting ${\tilde{A}}_{f}=\frac{1}{\lambda_{1}+\lambda_{2}\beta_{f}}$, ${\left|X_{\mathrm{MI}-\mathrm{SIN}R}\left(f\right)\right|}^{2}$ can be expressed as

(45) $${\left|X_{\mathrm{MI}-\mathrm{SIN}R}\left(f\right)\right|}^{2}=\max\left\{0,B(f)\left[{\tilde{A}}_{f}-D(f)\right]\right\}$$

It is worth noting that the spectrum response of the optimal waveform solution can also be obtained by the water-filling algorithm. Unlike the case without SINR constraints, it should be noted that the constant A changes to the variable ${\tilde{A}}_{f}$.

### 4.3. Waveform Design Using Relative Entropy

As in the case of the MI-based derivation, waveform optimization was considered in this work to maximize the KLD. Stein’s lemma [22] states that $P_{miss}$ (the probability of a miss, i.e., $1-P_{d}$) in the likelihood ratio test with $P_{fa}$ (the probability of a false alarm) decays at an exponential rate dictated by $D(p_{0}∥p_{1})$, i.e.,

(46) $$D(p_{0}∥p_{1})=\underset{N\to \infty }{\lim}\left[-\frac{1}{N}\log(1-P_{d})\right]$$

which implies that for any fixed $P_{fa}$, the maximization of the relative entropy leads to an asymptotic maximization of $P_{d}$. According to the probability of detection $P_{d}=Q\left[Q^{-1}(P_{fa})-\sqrt{d_{\mathrm{LMP}}^{2}}\right]$, to maximize the detection performance with respect to the transmitted signal, we only need to maximize $d_{\mathrm{LMP}}^{2}$. As a result, the maximization of the relative entropy is equal to the maximization of the deflection coefficient $d_{\mathrm{LMP}}^{2}$ of the locally most powerful (LMP) detector. Thus, the findings of this study were consistent with the findings in the study in [19]. In the presence of clutter, the expression of the approximate KLD based on the signal model in (4) is shown as

(47) $$\mathrm{KLD}\left({\left|X(f)\right|}^{2}\right)=\int_{W}\ln\left[1+\frac{\sigma_{h}^{2}(f){\left|X(f)\right|}^{2}}{P_{c}(f){\left|X(f)\right|}^{2}+\sigma_{n}^{2}}\right]\;-\frac{\sigma_{h}^{2}(f){\left|X(f)\right|}^{2}}{\sigma_{h}^{2}(f){\left|X(f)\right|}^{2}+P_{c}(f){\left|X(f)\right|}^{2}+\sigma_{n}^{2}}df$$

#### 4.3.1. Without SINR Constraints

The goal of this work was to describe the designed optimal transmitted waveform when the transmitted energy is fixed to the level $E_{x}$, i.e.,

(48) $$\begin{array}{l} \underset{{\left|X(f)\right|}^{2}}{\max}\;\int_{W}\ln\left[1+\frac{\sigma_{h}^{2}(f){\left|X(f)\right|}^{2}}{P_{c}(f){\left|X(f)\right|}^{2}+\sigma_{n}^{2}}\right]\;-\frac{\sigma_{h}^{2}(f){\left|X(f)\right|}^{2}}{\sigma_{h}^{2}(f){\left|X(f)\right|}^{2}+P_{c}(f){\left|X(f)\right|}^{2}+\sigma_{n}^{2}}df\\ s.t.\;\int_{W}{\left|X(f)\right|}^{2}\;df\leq E_{x} \end{array}$$

It is difficult to solve the above optimization problem directly due to the concave objective function. In this work, we used the Lagrangian multiplier technique, yielding a solution in the form of

(49) $$\mu ({\left|X(f)\right|}^{2},\lambda )=\ln\left[1+\frac{\sigma_{h}^{2}(f){\left|X(f)\right|}^{2}}{P_{c}(f){\left|X(f)\right|}^{2}+\sigma_{n}^{2}}\right]-\;\frac{\sigma_{h}^{2}(f){\left|X(f)\right|}^{2}}{\sigma_{h}^{2}(f){\left|X(f)\right|}^{2}+P_{c}(f){\left|X(f)\right|}^{2}+\sigma_{n}^{2}}-\lambda ({\left|X(f)\right|}^{2}-E_{x})$$

Let us apply the first derivative to $\mu ({\left|X(f)\right|}^{2},\lambda )$ with respect to ${\left|X(f)\right|}^{2}$; by setting it to zero, we obtain

(50) $$\frac{\partial \mu ({\left|X(f)\right|}^{2},\lambda )}{\partial {\left|X(f)\right|}^{2}}=\frac{\sigma_{h}^{4}(f)\sigma_{n}^{2}{\left|X(f)\right|}^{2}}{{\left[\sigma_{h}^{2}(f){\left|X(f)\right|}^{2}+P_{c}(f){\left|X(f)\right|}^{2}+\sigma_{n}^{2}\right]}^{2}\left[P_{c}(f){\left|X(f)\right|}^{2}+\sigma_{n}^{2}\right]}-\lambda =0$$

By solving (50), we obtain ${\left|X_{\mathrm{KLD}-\mathrm{NoSINR}}\left(f\right)\right|}^{2}$. By substituting ${\left|X_{\mathrm{KLD}-\mathrm{NoSINR}}\left(f\right)\right|}^{2}$ into constraint $\int_{W}{\left|X(f)\right|}^{2}\;df=E_{x}$, we obtain λ. This optimization procedure was obtained using an iterative water-filling method [35].

#### 4.3.2. With SINR Constraints

As in the case of MI-based models, under the SINR and energy constraints, the radar waveform design problem can be formulated as the following optimization problem:

(51) $$\begin{array}{l} \underset{{\left|X(f)\right|}^{2}}{\max}\;\int_{W}\ln\left[1+\frac{\sigma_{h}^{2}(f){\left|X(f)\right|}^{2}}{P_{c}(f){\left|X(f)\right|}^{2}+\sigma_{n}^{2}}\right]\;-\frac{\sigma_{h}^{2}(f){\left|X(f)\right|}^{2}}{\sigma_{h}^{2}(f){\left|X(f)\right|}^{2}+P_{c}(f){\left|X(f)\right|}^{2}+\sigma_{n}^{2}}df\\ s.t.\;\int_{W}{\left|X(f)\right|}^{2}\;df\leq E_{x}\\ \;\;\;\;\int_{W}\beta_{f}{\left|X(f)\right|}^{2}\;df\leq {\mathrm{SIN}R}_{0} \end{array}$$

We can obtain the optimal waveform by a similar method. The Lagrangian of the optimization in Equation (51) is [36]

(52) $$\mu ({\left|X(f)\right|}^{2},\lambda_{1},\lambda_{2})=\ln\left[1+\frac{\sigma_{h}^{2}(f){\left|X(f)\right|}^{2}}{P_{c}(f){\left|X(f)\right|}^{2}+\sigma_{n}^{2}}\right]-\;\frac{\sigma_{h}^{2}(f){\left|X(f)\right|}^{2}}{\sigma_{h}^{2}(f){\left|X(f)\right|}^{2}+P_{c}(f){\left|X(f)\right|}^{2}+\sigma_{n}^{2}}-(\lambda_{1}+\lambda_{2}\beta_{f}){\left|X(f)\right|}^{2}$$

Taking the partial derivative of $\mu ({\left|X(f)\right|}^{2},\lambda_{1},\lambda_{2})$ with respect to ${\left|X(f)\right|}^{2}$ and setting it to zero, we obtain

(53) $$\frac{\mu ({\left|X(f)\right|}^{2},\lambda_{1},\lambda_{2})}{\partial {\left|X(f)\right|}^{2}}=\frac{\sigma_{h}^{4}(f)\sigma_{n}^{2}{\left|X(f)\right|}^{2}}{{\left[\sigma_{h}^{2}(f){\left|X(f)\right|}^{2}+P_{c}(f){\left|X(f)\right|}^{2}+\sigma_{n}^{2}\right]}^{2}\left[P_{c}(f){\left|X(f)\right|}^{2}+\sigma_{n}^{2}\right]}-(\lambda_{1}+\lambda_{2}\beta_{f})=0$$

By solving (53), we obtain ${\left|X_{\mathrm{KLD}-\mathrm{SIN}R}\left(f\right)\right|}^{2}$. The corresponding experimental results are presented in Section 4.

### 4.4. SINR Value Range

As shown in the expressions of (14) and (17), the value of MI is between the values of XINR and SINR, and the value of KLD is between 0 and the J-divergence value. Therefore, the MI- and KLD-based optimal transmitted waveforms under the SINR constraint should consider the SINR threshold. In other words, we care more about how the designed transmitted waveform based on the MI and KLD is affected by the range value of the SINR.

Taking the derivatives of MI and XINR with respect to f, we obtain

(54) $$\mathrm{MI}{\left(f\right)}^{\prime }=\ln\left[1+\mathrm{SIN}R(f)\right]$$

(55) $$\mathrm{XINR}{\left(f\right)}^{\prime }=\frac{\mathrm{SIN}R(f)}{1+\mathrm{SIN}R(f)}$$

If $\mathrm{SIN}R(f_{k})\geq 0$ always exists for each frequency f∈W, then $\mathrm{MI}{\left(f\right)}^{\prime }\geq 0$ and $\mathrm{XINR}{\left(f\right)}^{\prime }\geq 0$ can be derived, guaranteeing that the MI and the XINR monotonically increase with the SINR. First, the case of the MI-based waveform design with no energy constraints and SINR tending to infinity was considered, where the MI can become infinity. However, given the limited energy, the MI becomes bounded, i.e., ${\mathrm{SIN}R}_{0}\leq \mathrm{SIN}R\left({\left|X_{\mathrm{MI}-\mathrm{NoSINR}}\left(f\right)\right|}^{2}\right)$.

Similarly, let SINR tend to negative infinity; the MI cannot become infinitely small under energy constraints and is greater than the maximum value of the XINR. Considering the energy constraint, the XINR-based radar waveform design problem can be formulated as the following optimization problem:

(56) $$\begin{array}{l} \underset{{\left|X(f)\right|}^{2}}{\max}\;\int_{W}\frac{\sigma_{h}^{2}(f){\left|X(f)\right|}^{2}}{\sigma_{h}^{2}(f){\left|X(f)\right|}^{2}+P_{c}(f){\left|X(f)\right|}^{2}+\sigma_{n}^{2}}\;df\\ s.t.\;\int_{W}{\left|X(f)\right|}^{2}\;df\leq E_{x} \end{array}$$

The Lagrangian multiplier technique is utilized here, and the objective function can be yielded as

(57) $$\mu ({\left|X(f)\right|}^{2},\lambda )=\frac{\sigma_{h}^{2}(f){\left|X(f)\right|}^{2}}{\sigma_{h}^{2}(f){\left|X(f)\right|}^{2}+P_{c}(f){\left|X(f)\right|}^{2}+\sigma_{n}^{2}}-\lambda ({\left|X(f)\right|}^{2}-E_{x})$$

Taking the partial derivative of $\mu ({\left|X(f)\right|}^{2},\lambda )$ with respect to ${\left|X(f)\right|}^{2}$ and setting it to zero, we obtain

(58) $${\left|X_{\mathrm{XINR}-\mathrm{NoSINR}}\left(f\right)\right|}^{2}=\max[0,\frac{\sqrt{\frac{P_{n}(f)\sigma_{h}^{2}\left(f\right)}{\lambda }}-P_{n}(f)}{P_{c}(f)+\sigma_{h}^{2}\left(f\right)}]$$

Since the MI is always greater than the XINR, the lower bound of the SINR should satisfy ${\mathrm{SIN}R}_{0}\geq \mathrm{SIN}R\left({\left|X_{\mathrm{XINR}-\mathrm{NoSINR}}\left(f\right)\right|}^{2}\right)$.

Therefore, the MI-based radar waveform design problem under the energy constraint has an SINR value range that should satisfy

(59) $$\mathrm{SIN}R\left({\left|X_{\mathrm{XINR}-\mathrm{NoSINR}}\left(f\right)\right|}^{2}\right)\leq {\mathrm{SIN}R}_{0}\leq \mathrm{SIN}R\left({\left|X_{\mathrm{MI}-\mathrm{NoSINR}}\left(f\right)\right|}^{2}\right)$$

Thus, our results indicated that, outside the interval of expression (59), SINR had no effect on maximizing MI under the energy constraints.

Like the method for maximizing MI, the KLD-based radar waveform design problem under the energy constraint presented an SINR value range that should satisfy

(60) $$0\leq {\mathrm{SI}\tilde{N}R}_{0}\leq \mathrm{SIN}R\left({\left|X_{\mathrm{KLD}-\mathrm{NoSINR}}\left(f\right)\right|}^{2}\right)$$

Additionally, outside the interval of expression (60), SINR exhibited no effect on maximizing KLD under the energy constraints.

## 5. Simulation and Results

To maximize radar detection performance, several simulation experiments are discussed herein to verify the validity of the proposed radar waveform design method based on the MI and KLD in the presence of clutter, considering not only the energy constraints but also the SINR constraints. Consider an extended target in the radar surveillance region with a known impulse response, and suppose the signal frequencies are between 0 MHz and 1 MHz and the sampling frequency is fs=2 to satisfy the Shannon sampling theorem. The noise variance is $\sigma_{n}^{2}=0.1$ and the energy constraint is $E_{x}=1$. For comparison, the same simulation scheme is performed for situations in the absence of clutter.

### 5.1. MI-Based Waveform and Value Range

**Figure 1:**
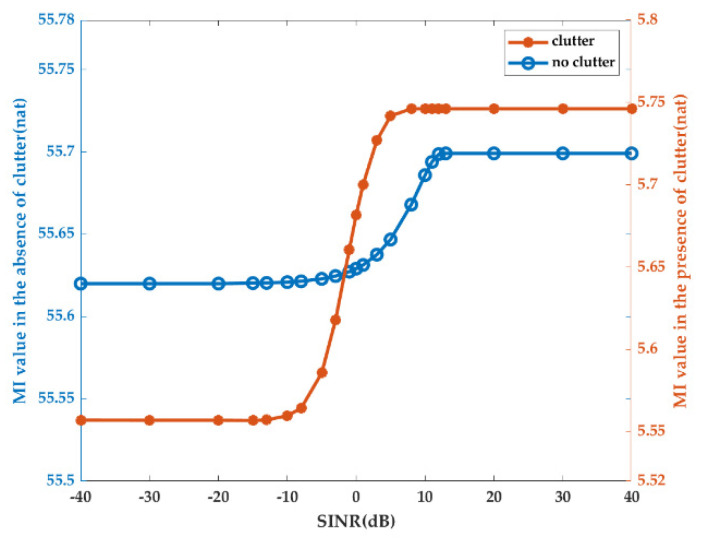
The maximum mutual information (MI) value versus signal-to-interference-plus-noise ratio (SINR) in the presence or absence of clutter.

The result in (14) was validated, while the maximization of MI (37) was acquired by waveform ${\left|X_{\mathrm{MI}-\mathrm{SIN}R}\left(f\right)\right|}^{2}$ with different SINRs. ${\left|X_{\mathrm{MI}-\mathrm{SIN}R}\left(f\right)\right|}^{2}$ is the optimal waveform under energy and SINR constraints. The maximization of MI in the absence of clutter was plotted (see the blue line in Figure 1) for comparison. Therefore, the maximum MI value versus SINR in the presence or absence of clutter is shown in Figure 1.

As seen in Figure 1, the maximum MI values both increased with increasing SINR in the presence or absence of clutter, as verified by Equation (54). Moreover, the SINR value range for the increasing MI was −10 dB to 8 dB in the presence of clutter and −5 dB to 13 dB in the absence of clutter, consistent with our analysis (59). Thus, depending on the change in optimal waveforms ${\left|X_{\mathrm{XINR}-\mathrm{NoSINR}}\left(f\right)\right|}^{2}$ and ${\left|X_{\mathrm{MI}-\mathrm{NoSINR}}\left(f\right)\right|}^{2}$, the SINR value range was affected by clutter and moved to the left on the coordinate axis. Additionally, outside the interval of expression (59), SINR had no effect on maximizing MI under the energy constraints. The orange line in Figure 1 shows that the MI value change interval was limited from 5.562 to 5.748 in the presence of clutter, greater than the value change interval in the absence of clutter. In addition, the gap between the maximum and minimum values of MI kept changing with the variation in the clutter spectrum.

**Figure 2:**
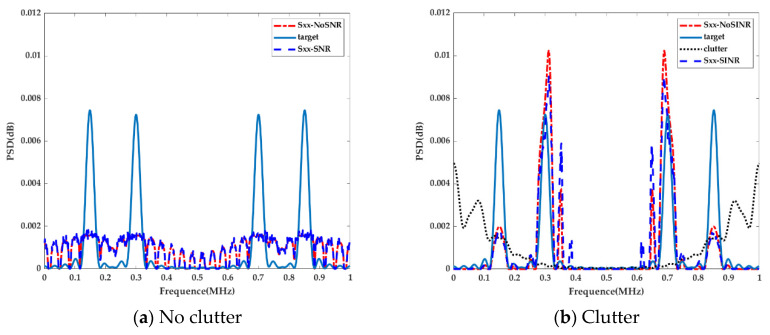
MI-based radar waveform design.

To verify the design of the optimal waveform given in (32) and (37), we set ${\mathrm{SIN}R}_{0}=1\mathrm{dB}$ and let the waveform energy normalization value vary. The power allocation strategy in the presence or absence of clutter is presented in Figure 2.

For both MI-based optimal radar waveform design strategies in the presence or absence of clutter, the transmitted energy allocation was determined by the target spectra and the clutter spectra. As Figure 2a shows, the MI-based optimized waveform (see the red line Sxx-NoSNR) without the SINR constraint in the absence of clutter presented a large value of power spectral density at the frequencies, whereas the target spectrum response also showed a larger value and obtained the maximum matching with the target; hence, the radar achieved the best detection performance. Moreover, for the extended random target case, the MI-based waveform distributed the energy over all bands. The SINR constraint could then be considered. As a result that $\beta_{f}$ in (39) is a constant in the absence of clutter and only one constraint among the energy and SINR constraints can work, the optimized waveform (see the blue line Sxx-SNR) exhibited slight fluctuations on the basis of Sxx-NoSNR. On the other hand, the MI-based waveform basically filled the dominant frequency band in the presence of clutter, as presented in Figure 2b. Note that the optimum transmitted waveform was different from the optimum transmitted waveform created in the absence of clutter. To maximize the MI between the target response and received waveform, the SINR constraint transmitted waveform distributed its limited energy over more bands of the target spectrum, unlike the case without the SINR constraint, where only a few dominant frequency components were chosen.

### 5.2. KLD-Based Waveform and Value Range

**Figure 3:**
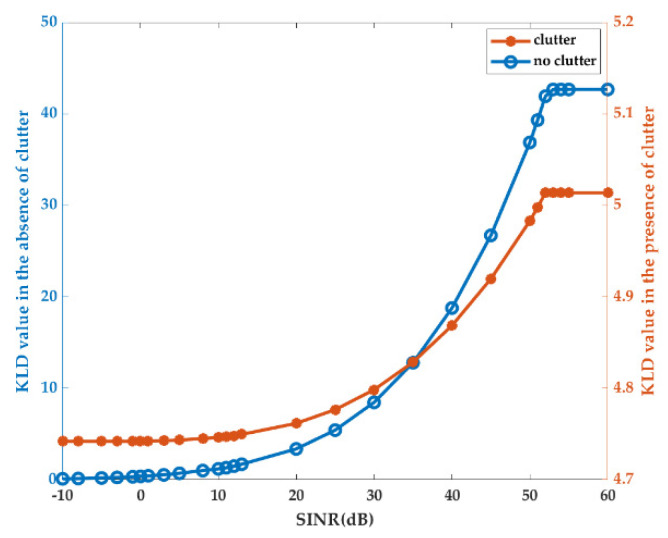
The maximum Kullback–Leibler divergence (KLD) value versus SINR in the presence or absence of clutter.

The second experiment was related to KLD-based radar waveform optimization. First, the result in (17) was validated, while maximization of the KLD (51) was acquired by waveform ${\left|X_{\mathrm{KLD}-\mathrm{SIN}R}\left(f\right)\right|}^{2}$ with different SINRs. The maximization of the KLD in the absence of clutter was plotted (see the blue line in Figure 3) for comparison. Therefore, the maximum KLD value versus the SINR in the presence or absence of clutter is shown in Figure 3.

Similarly, as seen in Figure 3, the maximum KLD values both increased with increasing SINR in the presence or absence of clutter, as verified by $\mathrm{SIN}R(f_{k})\geq 0$. Different from the maximum MI case, the SINR value range for increasing the KLD was 10 dB to 52 dB in the presence of clutter and −3 dB to 52 dB in the absence of clutter, consistent with our analysis (60). Another difference was that the KLD value change interval was limited from 4.74 to 5.01 in the presence of clutter, which was smaller than the value change interval in the absence of clutter. Additionally, the gap between the maximum and minimum values of the KLD kept changing with the variation in the clutter spectrum.

**Figure 4:**
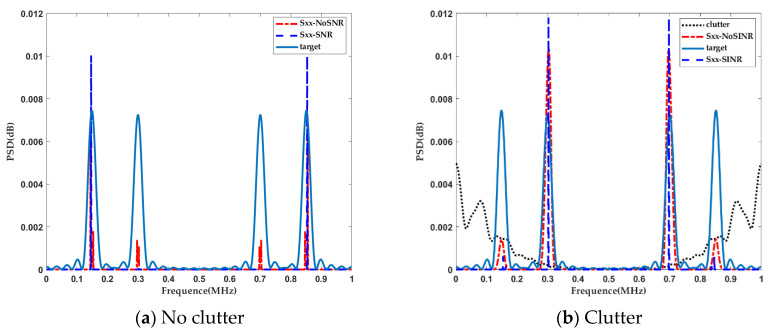
The KLD-based radar waveform design.

The KLD-based radar waveform design is shown in Figure 4. To better illustrate the changes in the optimized waveform in this case, we used the same simulation conditions as in Figure 2. At the same time, because the KLD waveform is concentrated at some frequency bins, to facilitate displaying the results, the obtained waveform based on maximizing the KLD was reduced in proportion.

It is important to observe that there was a significant difference in the power allocation strategy. Under the SINR constraint, the KLD-based waveform design split all the signal energies into two symmetric frequency bins, 0.15 MHz and 0.85 MHz, in the absence of clutter, where the ratio $\sigma_{h}^{2}(f)/\sigma_{n}^{2}$ was the maximum frequency, as shown in Figure 4a. In addition, the transmitted energy was distributed into frequency bins, 0.3 MHz and 0.7 MHz, in the absence of the SINR constraint. On the other hand, as seen in Figure 4b, under the SINR constraints, the energy distribution became more focused on the frequency bins of the maximum ratio $\sigma_{h}^{2}(f)/\left[\sigma_{n}^{2}+P_{c}(f)\right]$, which was the same as the results in [19].

### 5.3. Comparison of Detection Performance

**Figure 5:**
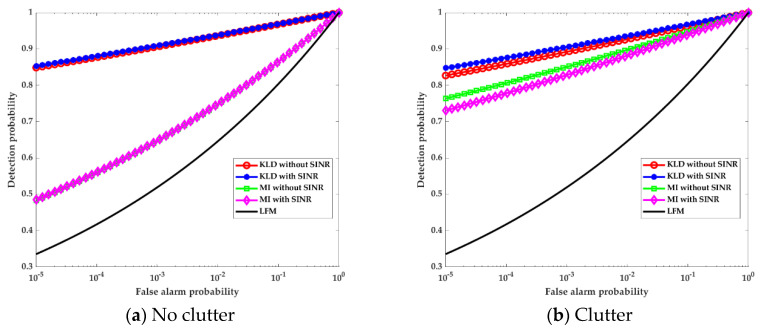
Detection probability curves corresponding to optimal waveforms and linear frequency modulated (LFM) waveforms.

To examine the detection performance of the MI- and KLD-based radar waveforms under the SINR constraint, we used the linear frequency modulated (LFM) waveform as a benchmark. Furthermore, the waveform optimal method in the absence of clutter was also considered for comparison. Therefore, the detection probability curves corresponding to the optimal waveforms and LFM waveforms in the presence or absence of clutter are shown in Figure 5.

As seen in Figure 5, the detection performance of the MI-based waveform was slightly worse than that of the KLD-based waveform, but better than that of the LFM waveform. Under the SINR constraint and in the presence of clutter, the detection performance of the KLD-based waveform improved, while the detection performance of the MI-based waveform decreased. However, under the SINR constraint and in the absence of clutter, the detection performance of the KLD- and MI-based waveforms improved. The waveform simulation results verified its correctness and effectiveness.

In summary, in the absence of clutter, the MI- and KLD-based waveform design methods under the SINR and energy constraints acquire higher detection performance than the same waveform design without the SINR constraint. In the presence of clutter, the MI-based waveform design method is suitable for target information extraction, while the KLD-based waveform design method is suitable for target detection. The results based on the SINR value range indicated that the optimal waveform should consider the trade-off between the MI, KLD, and SINR.

## 6. Conclusions

In this paper, we considered the optimal radar waveform design to detect a spectrum spread target in the presence of clutter. The optimal waveform was derived by maximizing the mutual information and the Kullback–Leibler divergence under the SINR constraint. We also compared the proposed optimized radar waveforms in the absence of clutter. Under the SINR constraint, the MI-based optimal signal waveform can make full use of the transmitted energy to target information extraction and put the signal energy in the frequency bin where the target spectrum is larger than the clutter spectrum. The KLD-based optimal signal waveform can make full use of the transmitted energy to detect the target and put the signal energy in the frequency bin with the maximum target spectrum. Numerical simulations showed that the KLD-based waveform generally outperforms the MI-based and LFM waveforms in terms of detection performance. Furthermore, the important relationships XINR≤MI≤SINR and 0≤KLD≤J-divergence connected the five design metrics of extended target waveform design and suggested that the SINR value range has a certain effect on MI- and KLD-based waveform designs. Future work should consider enforcing other constraints on the waveform.
